# The Clock and the Brain: Circadian Rhythm and Alzheimer’s Disease

**DOI:** 10.3390/cimb47070547

**Published:** 2025-07-15

**Authors:** Samaneh Ghorbani Shirkouhi, Ashkan Karimi, Seyed Sepehr Khatami, Ashkan Asgari Gashtrodkhani, Farzin Kamari, Morten Blaabjerg, Sasan Andalib

**Affiliations:** 1 Student Research Committee, School of Medicine, Shahroud University of Medical Sciences, Shahroud 36147-73943, Iran; samaneh.shirkouhi@shmu.ac.ir; 2 Department of Psychology, Centre for Vision Research, York University, Toronto, ON M3J 1P3, Canada; ashkan70@yorku.ca; 3 Department of Neurology, University of California Irvine, Irvine, CA 92617, USA; sskhatam@hs.uci.edu; 4 School of Medicine, Guilan University of Medical Sciences, Rasht 41937-13111, Iran; ashkan.asgari.gashtrodkhani@gmail.com; 5 Department of Neurophysiology, Institute of Physiology, Eberhard Karls University of Tuebingen, 72074 Tuebingen, Germany; farzin.kamari@uni-tuebingen.de; 6 Research Unit of Neurology, Department of Clinical Research, Faculty of Health Sciences, University of Southern Denmark, 5230 Odense, Denmark; morten.blaabjerg1@rsyd.dk; 7 Department of Neurology, Odense University Hospital, 5000 Odense, Denmark

**Keywords:** circadian rhythm, Alzheimer’s Disease, molecular mechanisms, cellular mechanisms

## Abstract

Alzheimer’s Disease (AD) is the most common type of dementia. The circadian system, which is controlled by the master clock in the Suprachiasmatic Nucleus (SCN) of the hypothalamus, is crucial for various physiological processes. Studies have shown that changes in the circadian rhythms can deteriorate neurodegenerative diseases. Changes in the SCN are associated with cognitive decline in AD. The cognitive impairments in AD, especially memory dysfunctions, may be related to Circadian Rhythm Disturbances (CRDs). Moreover, rhythmic expression of clock genes is disrupted in AD patients. There is a circadian pattern of inflammatory processes in AD, and dysregulation of core clock genes promotes neuroinflammation. The present narrative review addresses the intricate link between CRDs and AD, revisiting the relevant cellular and molecular mechanisms. The association between CRDs and AD highlights the need for further investigation of the underlying mechanisms.

## 1. Introduction

Alzheimer’s Disease (AD) is a progressive neurodegenerative disorder and the most common type of dementia without any curative therapy. AD starts with losses in recent memory and gradually progresses to long-term memory deficits and further affects other cognitive domains such as language, reasoning, conscious thought, behavior, and personality [1]. Besides the enlargement of brain ventricles [2], various brain regions become atrophic in AD. Primarily, the brain cortex is known to shrink during the disease, but alterations in the white matter are also visible [3]. The exact cause of AD has not yet been fully understood; however, the main theories suggest the role of Amyloid beta (Aβ) accumulations between neurons and neurofibrillary tangles (NFTs) of hyperphosphorylated tau protein inside neurons [4]. Intraneuronal accumulation of Aβ peptides starts prior to the extracellular amyloid plaques formation and tau NFTs [5]. Moreover, soluble Aβ also accumulates intracellularly, and then gradually changes to insoluble Aβ, and consequently impairs cellular functions [6]. Other introduced features in AD are neuroinflammation, brain vascular changes, and synaptic and dendritic spine loss [7,8].

The circadian system, controlled by the master clock in the Suprachiasmatic Nucleus (SCN) of the hypothalamus, is crucial for various physiological processes, including sleep–wake cycles, hormone secretion, and body temperature. The SCN generates an approximately 24 h rhythm [9]. This rhythm is synchronized with external environmental cues, known as Zeitgebers, but can also operate independently of them. A Zeitgeber is any external or environmental signal that regulates the circadian rhythms of an organism [10]. The period of circadian rhythms can be changed by molecular and environmental factors while the amplitude remains the same. Vanderleest et al. studied the association of amplitude changes and photoperiod variations and suggested an SCN pathway contributing to period length independent of amplitude [11]. Light, a strong Zeitgeber, activates the SCN through light-sensitive retinal ganglion cells in the eye. Studies have shown that the impaired coordination between central and peripheral circadian clocks leads to an increased risk of obesity, metabolic disorders, and dementia [12,13].

Studies have illustrated that changes in circadian rhythms can lead to neurodegenerative diseases such as AD. For instance, changes in the SCN are associated with cognitive deterioration in AD. The cognitive impairment in AD, especially memory disturbances, may also be related to Circadian Rhythm Disturbances (CRDs). Therefore, understanding the role of the circadian rhythm and its underlying cellular and molecular mechanisms in AD is of crucial importance. The present narrative review focuses on the intricate link between CRDs and AD and revisits the relevant cellular and molecular mechanisms.

## 2. Effect of Aging on Circadian Rhythms in the Brain

The brain undergoes changes in volume and size during the natural aging process. This also includes a decrease in the number of cells in the SCN, which is the primary circadian pacemaker in mammals [14]. A decrease in the amplitude of the circadian rhythm and impairment in the quality of nocturnal sleep are the most prominent changes observed during aging [15,16]. In the elderly, changes in circadian rhythm can affect temperature regulation, perception, information processing, and general cognitive abilities [17]. It appears that age-related disruption in circadian rhythms is due to dysfunction of the SCN [18]. Sleep disturbance due to age-related CRDs can lead to more serious and widespread disorders, including cognitive dysfunction and neurodegenerative diseases, including AD [19]. Early signs of CRDs may be a potential biomarker for the diagnosis of AD. Preclinical (asymptomatic) AD was shown to be associated with rest-activity rhythm fragmentation [20]. A previous study showed that healthy older women with a reduction in circadian activity rhythm amplitude and robustness, and delayed rhythms increased odds of developing dementia and Mild Cognitive Impairment (MCI) [21]. An association between weakened rest-activity rhythms, such as overall rhythmicity in older women, and adverse cognitive outcomes has been reported [22]. Disrupted rest-activity rhythms in older men are also associated with greater cognitive decline [23]. Similarly, age-related changes in the circadian rhythms have been observed in aged rodents [24].

## 3. Evidence of CRDs in AD Patients

A study reported that 40% of the AD patients had a disruption in their sleep [25]. Sleep fragmentation, shorter bouts of Rapid Eye Movement (REM) sleep [26], and reduced slow-wave sleep percentage [27] have also been reported in patients with AD.

Beyond sleep–wake disturbances, AD patients exhibit abnormalities in the core circadian markers, including, but not limited to, altered timing of melatonin’s nocturnal peak [28] and reduced amplitude and phase delays in body temperature rhythms [29].

These CRDs have substantial clinical significance, representing a leading cause of caregiver burden and institutionalization [30], while also contributing to cognitive impairment through disrupted synaptic homeostasis and memory consolidation processes [31]. The presence and severity of CRDs could potentially predict faster rates of cognitive decline and disease progression [21]. It has been suggested that these disturbances may precede cognitive symptoms for several years, supporting their potential utility as early biomarkers of AD pathology [20].

## 4. Sex Differences in CRDs in AD

Circadian rhythms in mammals are dependent on sex. The SCN and other brain regions contain androgen and estrogen receptors that regulate activity rhythms differently in males and females [32]. In C57BL/6N male and female mice, circadian misalignment was induced by a chronic jet lag (a temporary condition occurring when the body’s internal clock becomes misaligned with the local time after traveling across multiple time zones) shift schedule [32]. Significant sex-based differences were observed in circadian clock organization and metabolism. Testosterone plays a key role in maintaining the circadian clock in males [32]. A study examined how chronic CRD could contribute to the pathology and rate of progression of AD in an AD mouse model [33]. Female animals showed a greater adaptability to CRD. In addition, CRD worsened the performance of male animals in the Morris water maze behavioral test, whereas no effect was observed in females. Finally, the authors concluded that exposure to chronic CRD impairs circadian behavioral patterns and cognitive phenotypes in a sex-dependent manner [33]. Another study assessed fractal motor activity regulation (FMAR) in 178 cognitively normal participants who underwent 7 to 14 days of home actigraphy [34]. Amyloid Imaging-Pittsburgh compound B (PiB) and Cerebrospinal Fluid (CSF) phosphorylated-tau181 to Aβ42 ratio were used to assess the presence of preclinical AD pathology. Alterations in daytime FMAR appear to manifest in women early in preclinical AD [34].

## 5. Molecular Mechanisms Linking AD and Circadian Rhythm

### 5.1. Expression of Clock Genes in AD

**Figure 1:**
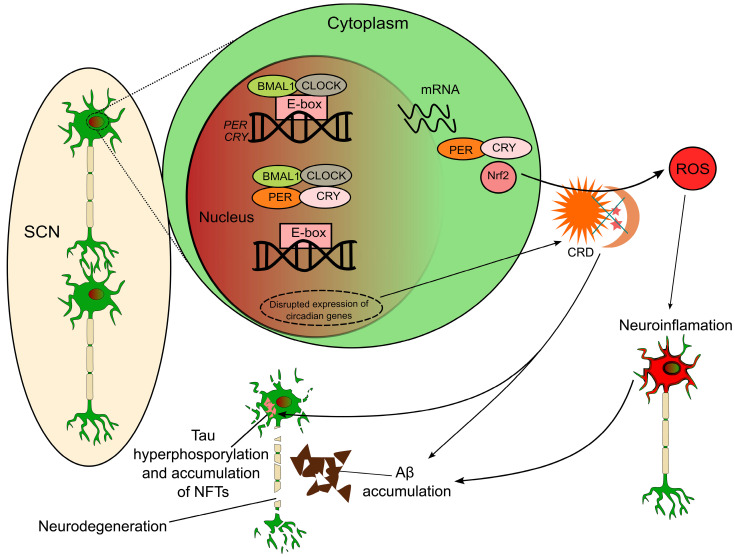
The cellular and molecular mechanisms involved in the association of the circadian clock and Alzheimer’s Disease (AD). BMAL1:CLOCK complex binds to Enhancer Box (E-Box) element in the nucleus of the Suprachiasmatic Nucleus (SCN). BMAL1 and CLOCK drive the expression of *PER* and *CRY*. PER and CRY form a complex in the cytoplasm, and this complex is transferred to the nucleus. PER and CRY inhibit the transcriptional activity of the BMAL1:CLOCK complex after their translation and nuclear accumulation. PER and CRY protein levels reduction decreases the suppression of BMAL1:CLOCK activity, and a new cycle begins. In AD patients, the disrupted expression of clock genes leads to Circadian Rhythm Disturbance (CRD), which exacerbate Amyloid beta (Aβ) accumulation between neurons and Neurofibrillary Tangles (NFTs) of hyperphosphorylated tau protein within neurons. In addition, nuclear factor erythroid 2-related factor 2 (Nrf2) activation leads to upregulation of *Cry2*. It has been shown that in the absence of *Bmal1*, Nrf2 activity is reduced, which leads to a decreased antioxidant response and a subsequent rise in Reactive Oxygen Species (ROS). ROS leads to neuroinflammation. Neuroinflammation can accelerate Aβ accumulation. Abbreviations: AD, Alzheimer’s Disease; BMAL1, basic helix-loop-helix ARNT like 1; CLOCK, Circadian Locomotor Output Cycles Kaput; E-Box, Enhancer Box; PER, Period; CRY, Cryptochrome; SCN, Suprachiasmatic Nucleus; Aβ, Amyloid beta; CRD, Circadian Rhythm Disturbance; NFTs, Neurofibrillary Tangles; Nrf2, nuclear factor-erythroid 2 related factor 2; ROS, Reactive Oxygen Species; mRNA, messenger ribonucleic acid.

The molecular structure of the circadian clock in mammals, SCN, consists of two interlocking Transcription/Translation Feedback Loops (TTFLs), which are directed by two activators [Circadian Locomotor Output Cycles Kaput (*CLOCK*) and basic helix-loop-helix ARNT like 1 (*BMAL1*)] and two repressors [Period (*PER*) and Cryptochrome (*CRY*)] [35]. Transcription of *BMAL1* and *CLOCK* genes leads to the heterodimerization of the BMAL1:CLOCK complex in the cytoplasm. This complex is transferred to the nucleus and binds to canonical Enhancer Box (E-Box) sequences or noncanonical E-Boxes of clock-regulated genes. BMAL1 and CLOCK drive the expression of *PER* and *CRY.* PER and CRY proteins form a complex in the cytoplasm, and this complex is transferred to the nucleus. PER and CRY proteins inhibit the transcriptional activity of the BMAL1:CLOCK complex after their translation and nuclear accumulation. PER and CRY protein levels reduction decreases the suppression of BMAL1:CLOCK activity, and a new cycle begins [36]. Figure 1 illustrates the cellular and molecular mechanisms involved in the association of the circadian clock and AD.

Neuronal dysfunction has also been observed in the SCN of patients with AD [14,37]. Similarly, signs of degeneration, including increased glia/neuron ratio in the SCN tissue of postmortem AD patients, have been detected [38]. Moreover, in AD patients, melatonin levels are reduced [39]. These changes could potentially contribute to disruptions in circadian gene expression and hence CRDs.

It has been shown that rhythmic expression of clock genes was lost in both preclinical and clinical AD patients [40]. A study by Luo et al. demonstrated that sleep deprivation (SD) upregulated *Cry2* in the hippocampus of AD mice by decreasing cytokine-inducible SH2-containing protein (CISH)-mediated transcription factor Signal Transducer and Activator of Transcription 1 (STAT1) phosphorylation, which resulted in synaptic dysfunction [41]. Oyegbami et al. showed a blunted effect of *Cry1* and *Cry2* gene expression in the medulla/pons of the AD mouse model [38]. In a study in the AD mouse model with disrupted circadian clock function via *Bmal1* gene deletion, disruption of daily hippocampal Interstitial Fluid (ISF) Aβ oscillations, and increased amyloid plaque accumulation were observed [42].

A recent preclinical study has shown a close relationship between sleep–wake cycle parameters and circadian clock gene expression levels, especially within the SCN and hippocampus in 2-month-old (plaque-free stage) and 10-month-old (plaque-burdened stage) AD mouse models [43]. The result showed that CRDs preceded Aβ deposition.

Longitudinal studies can help to determine whether clock gene disruptions are a cause or consequence of AD pathology. By observing over time, researchers can follow the events leading to neurodegeneration. Assessing circadian gene expression along with Aβ and tau accumulation in animal models can help to find which one precedes the other.

### 5.2. Circadian Clock Gene Polymorphisms Associated with AD

Genetic factors have a significant role in both AD pathogenesis and circadian rhythm regulation. There is evidence of polymorphisms in several genes involved in circadian clock function that may influence AD progression and the manifestation of CRDs.

The clock genes encode a transcription factor central to circadian rhythm generation and contain several Single Nucleotide Polymorphisms (SNPs) associated with AD. Studies identified the higher prevalence of the C allele of the *CLOCK* gene for rs4580704 SNP [44] and also a higher prevalence of the rs1554483 G allele [45] in AD patients compared to controls in the Chinese population.

A significant association between the rs3027178 SNP of the *PER1* gene and AD, where the G allele was protective against AD has been reported [46]. It was proposed that this polymorphism can influence the expression of genes that can be relevant for AD, including Vesicle-Associated Membrane Protein 2 (VAMP2) in the hypothalamus and CST Telomere Replication Complex Component 1 (CTC1) protein across several tissues. Xiang et al. showed an increased prevalence of five-repeat homozygotes of *PER3* length in AD patients compared to controls [47].

*BMAL* genes contain variants that have been identified in AD patients. Chen et al. found a higher prevalence of TT genotypes *ARNTL* (*BMAL1*) gene rs2278749 SNP in AD patients compared to that in controls [48]. Qing-Xiu et al. also showed a significantly higher prevalence of C allele and CC genotypes of *ARNTL2* (*BMAL2*) gene rs2306074 SNP in AD patients compared to that in controls [49].

Yang et al. reported that the prevalence of the C allele of the *CLOCK* gene 3111T/C SNP in AD patients was significantly higher than that in control subjects in the Chinese population [22]. A study in the Chinese population showed that the prevalence of the A allele of the *CLOCK* gene rs4864548 SNP in AD patients was significantly higher than that in control subjects [50].

In the Chinese population, a significant difference was detected in *ARNTL* (*BMAL1*) gene rs900147 SNP between AD and control subjects regarding the genotypic distribution [51]. This study also showed that G allele carriers have a significantly higher risk of developing AD and amnestic MCI than other carriers.

A study of the Mexican population reported that *PER3* gene rs228697 SNP showed a nine-fold increased risk for CRDs in AD patients [52]. It was proposed that this variant may contribute to circadian disruption, leading to sleep disturbances in AD patients. This, in turn, exacerbates Aβ pathology and may accelerate neurodegeneration. The study also mentioned that the risk of CRDs may be higher in Apolipoprotein E4 (APOE ε4) carrier patients because of a potential interaction between PERIOD protein and *APOE*.

For *CLOCK* gene rs4580704 [44], rs1554483 [45], rs4864548 [50] SNPs, *BMAL1* gene rs2278749 [48], and *BMAL2* gene rs2306074 [49] SNPs, the authors argued that they might contribute to increasing AD risk through dysregulation of metabolic pathways, potentially overlapping with APOE ε4-associated mechanisms, and possibly exacerbating neurodegeneration via impaired glucose [53] and lipid [54] metabolisms, and an increase in metabolic syndrome risk [55].

These genetic associations provide insight into shared biological pathways between CRDs and AD pathogenesis, while suggesting that genotyping of clock-associated gene polymorphisms may help identify high-risk individuals and provide guidance for personalized chronotherapeutic interventions. 

Table 1 summarizes some polymorphisms in circadian clock genes that affect AD.

### 5.3. Impact of Amyloid-Beta and Tau on Circadian Regulation

Vasopressin is mostly synthesized in the supraoptic nucleus (SON) and paraventricular nucleus (PVN) of the hypothalamus. It acts as a hormone and neurotransmitter. Vasopressin plays a key role in the homeostasis of water and electrolytes in the body and is a vasoconstrictor [56]. It is also a modulator of the stress response and vital autonomic functions, including body temperature, behavior, and memory [57]. Neurotensin (NT) is a 13-amino-acid peptide that was originally isolated from bovine hypothalami in 1973 [58]. NT is found in the central nervous system and gastrointestinal tract [59]. The association between NT as a neuromodulator and dopamine in the nervous system has been well known [59]. A post-mortem study showed that patients with AD compared to controls exhibited a significant decrease in vasopressin and NT and a corresponding increase in the Glial Fibrillary Acidic Protein (GFAP)-stained astrocyte to Nissl-stained neuron ratio in the SCN [60]. Additionally, Kang et al. found that ISF Aβ levels significantly increase during acute SD and with orexin infusion, whereas Aβ levels decreased following administration of a dual orexin receptor antagonist [61].

It has been shown that Aβ administration in the SCN can cause CRDs [62]. Aβ accumulation has been shown to impair melatonin receptor signaling in the SCN and pineal gland [28]. A post-mortem study showed reduced melatonin receptors (MT1 and MT2) expression in the pineal gland and the occipital cortex of AD patients, which correlates with Aβ burden severity [63]. The downregulation of these receptors may contribute to the loss of melatonin’s chronobiotic effects in AD patients.

Pappolla et al. demonstrated that melatonin prevents Aβ aggregation rather than reversing the neuropathology in the clinical phases of AD [64]. They studied the effect of melatonin on the clearance of Aβ peptides through the lymphatic system. The study was performed on AD transgenic mouse models (Tg2576) treated with melatonin at the ages of 4 months and 15.5 months. Aβ (Aβ42, Aβ40) levels were measured in the lymph nodes, brain, plasma, and multiple tissues one week after the treatment in the treated and control groups. A remarkable reduction in Aβ42 and oligomeric Aβ40 levels in the brain and also a notable increase in soluble monomeric Aβ40 levels in the brain and cervical lymph nodes, which showed that melatonin increases Aβ lymphatic clearance system were seen. Regarding the anti-amyloid effects of melatonin, melatonin treatment initiation before the age of amyloid formation at the age of 4 months exhibited a greater response in comparison with 15.5 months [64]. The decreased melatonin levels may contribute further to compromising sleep quality and worsening circadian desynchronization.

A study investigating AD-related pathology and CRD in an Amyloid Precursor Protein with the Swedish mutation (APPSwe)-Tau (TAPP) mouse model, showed phase-delayed body temperature and locomotor activity with increases around the active-to-rest phase transition [65]. It was also shown that CRD and aggression coincide with hyperphosphorylated Tau (pTau) development in Lateral Parabrachial (LPB) neurons. These neurons, including those expressing dynorphin (LPB^dyn^), project to circadian structures and are affected by pTau. The ablation of LPB^dyn^ partially recapitulated the hyperthermia in this mouse model [65]. Another study investigating the relationship between the sleep–wake cycle impairment and the CSF AD biomarkers and CSF orexin concentrations in mild to moderate AD patients showed a correlation between tau proteins and orexin CSF levels and the sleep–wake cycle dysregulation [66].

Using tau-specific Positron Emission Tomography (PET) imaging, Lucey et al. demonstrated that regions known to be involved with AD progression showed associations with reduced slow-wave activity of non-REM (NREM) sleep in orbitofrontal, entorhinal, lingual, parahippocampal, and inferior parietal areas [67]. These findings indicate a relationship between regional tau pathology and specific sleep architecture changes important for memory consolidation.

### 5.4. Neuroinflammation and Oxidative Stress as Mediators of CRDs in AD

Oxidative stress, through hydrogen peroxide activity, has the potential to shift the phase of the circadian clock in the brain and other organs [68]. This phenomenon further depends on the time of the day and the dose of the oxidative stress produced. On the other hand, oxidative stress can regulate the expression of antioxidant genes by modulating nuclear factor-erythroid 2 related factor 2 (Nrf2), a transcription factor [69]. Nrf2 activation can further give rise to the upregulation of the repressor gene *Cry2* and lead to CLOCK-BMAL1 transcriptional activation, which are critical components of the circadian clock [69].

Not only is the circadian clock disrupted in AD, but another study also showed a bidirectional relationship between oxidative stress and disruptions of the circadian clock, resulting in the progression of cognitive decline in AD [70]. This study showed that environmental stress following the CRDs is related to elevated levels of Nicotinamide Adenine Dinucleotide Hydrogen (NADH) and decreased levels of Glutathione (GSH), which lead to the progression of cognitive deficits in AD.

In addition, a recent study has highlighted the molecular mechanisms of CRD associated with cognitive decline via neuroinflammation [71]. This study indicated that microglial activation and the subsequent production of cytokines led to a loss of neurogenesis and a reduction in synaptic proteins in the hippocampus. Furthermore, neuroinflammation induced by CRD was prevented by removing microglia in the hippocampus. Another study by Fonken et al. indicated that neuroinflammation has a potential effect on the expression of core circadian clock genes in the SCN, including *Bmal1*, *Per2*, and *Clock* [72]. Moreover, in the setting of circadian disruptions, elevated levels of proinflammatory cytokines, including Interleukin-1 beta (IL-1β) and Tumor Necrosis Factor-alpha (TNF-α), were detected [72]. In a study on mice, TNF-α influenced the expression of the D site of albumin promoter binding protein *(Dbp)*, a circadian clock gene, in the SCN, resulting in the prolongation of the rest period in the dark, i.e., the active phase for mice [73]. Additionally, TNF-α inhibits the activation of the E-Box, a DNA regulatory circadian gene expression element, through the CLOCK-BMAL1 complex. Increased IL-1β levels have been observed in macrophages lacking *Bmal1*, a crucial transcription factor that modulates the antioxidant transcription factor Nrf2 [74]. In the absence of *Bmal1*, Nrf2 activity is reduced, which leads to a decreased antioxidant response and subsequent rise in reactive oxygen species (ROS) (Figure 1). ROS further regulates Hypoxia-Inducible Factor 1-alpha (HIF-1α), a transcription factor that upregulates the production of IL-1β [74]. A study in an AD mouse model showed that neuroinflammation can accelerate Aβ deposition [75].

In another study on mice investigating the relationship between Interleukin-6 (IL-6) and circadian rhythms, it was found that mice lacking IL-6 showed altered expression of *Cry1*, Differentiated Embryo Chondrocyte 2 *(Dec2)*, and Nuclear Receptor Subfamily 1 Group D Member 2 (*Rev-erbβ)* [76]. This study demonstrated the role of IL-6 in regulating ultradian activity (shorter cycles that occur several times throughout the day), rest rhythmicity, and clock gene expression, which may contribute to CRDs in some neurodegenerative diseases, including AD.

In a study of the effect of Interferon gamma (IFN-γ) on rat SCN cultures and its effect on the expression of clock genes, it was found that cells exposed to IFN-γ showed decreased average spiking frequency and exhibited a higher level of irregular firing pattern in addition to a lower expression amplitude of *Per1* in SCN neurons [77]. This study demonstrates that IFN-γ may alter the circadian rhythms and sleep disturbances in the aging brain.

A study showed that exposure of different types of cells, including neurons, to Aβ and its fragments led to a slowing of the circadian rhythms through mitochondrial dysfunction [78]. In this study, different Aβ species, including Aβ1–42, Aβ1–40, Aβ1–28, Aβ34–42, Aβ15–25, and Aβ25–35, were used within the same concentration range. The results also showed that all Aβ species induce remarkable changes in circadian function similar to more neurotoxic Aβ1–42. Moreover, Aβ is linked to changes in circadian length compared to changes in amplitude. Additionally, Aβ induces mitochondrial Adenosine Triphosphate (ATP) depletion and reduced energy levels, elevated oxidative stress and ROS, and a decline in mitochondrial respiration, which results in the mitochondria’s inability to respond to elevated metabolic demand and subsequent CRDs.

It is worth mentioning that even though there are several studies [79,80,81] that have highlighted the important role of neuroinflammation and oxidative stress in CRD, further studies need to be conducted to understand the exact mechanisms behind this interaction due to its complexity. Some studies [70,82] have emphasized that this relationship is bidirectional. Hence, it is crucial to evaluate the causal direction of the link between CRD and neuroinflammation. These studies used animal models, including mice [73,76]. This could be a limitation. To evaluate this association, thus, future clinical trials need to be conducted to confirm it in clinical settings. The variation in therapeutic agents used to investigate these underlying pathways has led to a persistent knowledge gap regarding this relationship [83].

## 6. Bidirectional Relationship Between AD and Circadian Rhythms

Brain-derived Neurotrophic Factor (BDNF) is an important neurotrophic factor involved in neuronal plasticity and cognitive function. The reduced level of BDNF in brain tissue samples of AD patients indicates its pivotal role in the pathogenesis of AD [84]. The increased BDNF levels reduce the Aβ peptide production that raises the neurogenesis and cognitive and memory function in AD [85]. The circadian rhythms regulate BDNF expression and its neuroprotective outcomes [86]. BDNF promotes anti-amyloidogenic APP processing by increasing α-secretase activity, which prevents APP peptides from being processed by β-secretase and subsequent production of neurotoxic Aβ peptides [85]. CRDs, however, can promote cognitive impairment and the progression of AD by decreasing BDNF levels and increasing Aβ deposition in the hippocampus [87]. Aβ aggregations disrupt Tyrosine receptor kinase B (TrkB) axonal processing and retrograde transport that reduce BDNF retrograde signaling, which impair axonal transport and synaptic plasticity in Tg2576 [88].

From another perspective, sleep has a crucial function in the clearance and removal of brain neurotoxic waste products containing Aβ [89]; hence, SD is related to accelerate Aβ production and AD progression [90].

Circadian core clock genes are the fundamental parts of the circadian rhythms and are associated with the regulation of cellular metabolism, physiological homeostasis, inflammatory responses, and immunity [91]. As mentioned above, mutation or dysfunction in the expression of core clock genes such as *Bmal1* and *Clock* is related to different aspects of aging and neurodegeneration [92]. Targeted core clock *Bmal1* gene deletion enhances Aβ plaque formation [42]. Core clock *Bmal1* gene deletion also induces autonomous astrocyte activation and astrogliosis [93].

A randomized clinical trial investigating the effect of SD on the level of Aβ in the CSF of healthy middle-aged men revealed that SD, or prolonged wakefulness, interferes with a physiological reduction in CSF Aβ [94]. Shokri-Kojori et al. similarly showed that one-night SD led to an increased Aβ burden in the hippocampus and thalamus of healthy individuals, suggesting that sleep, among other factors, could influence Aβ clearance in the human brain [95].

In addition, Aβ accumulation damages SCN neurons, further disrupting sleep quality in a vicious cycle. The glymphatic system, which facilitates Aβ clearance, operates during natural sleep or anesthesia and increases the Aβ clearance by 60% compared to wakefulness [89]. It is suggested that the enlarged cortical interstitial space during sleep and the consequent increase in CSF-ISF exchange and transport of waste products through the glymphatic system could be the probable mechanism beyond the effect of sleep on the enhancement of Aβ clearance [89].

Lee et al. performed a study on the effect of body posture on the glymphatic system for the clearance of waste products, including Aβ from the brain [96]. They studied three different body postures in anesthetized rodents’ brains in supine, prone, and lateral positions to determine the CSF-ISF exchange rates. The results showed that the removal of waste products consisting of Aβ through the glymphatic transport was highest and most efficient in the lateral position, which represents the sleep state, and also was the lowest in the prone position, which represents the wake state.

In a study, the fruit fly *Drosophila* was used as a model of AD to find out the interaction between sleep and Aβ and the development of AD [97]. The result indicated that sleep deprivation increases the spontaneous neuronal action potential (AP) firing rate and also impairs the voltage-gated K^+^ channels, which causes neuronal hyperexcitability and further Aβ accumulation. In turn, Aβ was shown to induce hyperexcitability of neurons and further result in decreased and fragmented sleep. These findings showed a bidirectional relationship between sleep and Aβ burden to the enhancement of neuronal excitability and also the positive feedback loop mechanism beyond sleep, Aβ accumulation, and the hyperexcitation of neurons in the development of AD. The authors also suggested that anti-epileptic drugs could be a therapeutic agent for the prevention and slowing of the progression of AD by suppressing the neuronal hyperexcitation [97].

Evidence from an earlier study indicated a ~90% increase in ISF tau during normal wakefulness, compared to sleep, and a ~100% increment in SD in the AD mouse model. More than 50% increase in human CSF during SD was also observed [98].

## 7. Peripheral Circadian Clock and AD

Investigation of the rhythmicity in clock gene and protein expression in cells and tissue of the whole body in mammals has demonstrated that cells other than the SCN also contain endogenous circadian oscillators [99,100]. Lesions of the SCN in mPer2 (Luciferase) knockin mice did not eliminate circadian rhythms in peripheral tissues, but instead caused phase desynchrony among the tissues of individual animals [99]. CRDs may indirectly contribute to chronic gut microbiota (GM) dysbiosis through altering eating habits and metabolism. The interaction between CRDs and GM dysbiosis may work synergistically, promoting neuroinflammation and Aβ deposition, thereby playing a critical role in the onset and progression of AD [101].

## 8. Therapeutic Implications

### 8.1. Potential Interventions Targeting Circadian Rhythms

#### 8.1.1. Light Therapy

Recently, light therapy has been proposed as a promising, non-pharmacological, and non-invasive method for modulating CRD and memory impairments in animal models and human studies.

A rather recent study indicated that transcranial photostimulation (PS) in an AD mouse model facilitated the removal of Aβ from the brain, which was more prominent at night than during the day or wakefulness [102]. This study showed improvements in memory and a decrease in Aβ levels in the brains of AD mice following night-time PS. This result may be due to circadian oscillations in Aβ levels in the brain, as Aβ clearance is more efficient during sleep [61].

A randomized controlled trial study investigated the effects of a blue-enriched light therapy on AD patients, measured by Dim Light Melatonin Onset (DLMO) [103]. This study indicated that light therapy resulted in a phase shift in melatonin secretion, an improvement in sleep quality, an enhancement in cognitive performance, and a reduction in the time between DLMO and falling asleep.

#### 8.1.2. Therapeutic Targeting of Casein Kinase 1δ/ε

An interesting study indicated that blocking Casein Kinase 1 delta/epsilon (CK1δ/ε), which is a clock regulator and overexpressed in AD, modulates the hippocampal proteome in an AD mouse model, notably improving the expression of proteins involved in synaptic plasticity and APP processing [104]. This study showed that CK1δ/ε inhibition enhanced working memory performance and reduced behavioral CRDs in AD, for which this therapeutic approach may be considered a potential intervention targeting both CRDs and cognitive deficits in AD. Even though this study illustrated that CK1δ/ε inhibitor has the ability to modulate CRDs in the AD mouse model [104], a rat model study pointed out concerns regarding specificity, off-target effects, and timing of its administration in rats [105]. CK1δ/ε inhibitors may affect other signaling pathways beyond circadian regulation, including Wnt signaling [106], cell cycle progression [107], and DNA regulation, which may lead to toxicity and side effects. Administration of CK1δ/ε inhibitors at different time points of the day may result in various effects, and chronic use of these agents may have cumulative effects, highlighting the importance of both the dose and the timing of CK1δ/ε inhibitors administration [105].

### 8.2. Chronotherapeutics

Chronotherapeutics is the science of considering the delivery of drugs and therapeutic methods based on the inherent activity of each disease over a specific period of time. The aim of chronotherapy is to synchronize treatment or intervention with the inherent timing of the disease [108].

In a study on an AD mouse model, Time-Restricted Feeding (TRF) resulted in increased production of β-Hydroxybutyrate and decreased levels of blood glucose [109]. According to this study, TRF enhanced total sleep and sleep-phase consolidation and decreased fragmentation and agitation.

The investigation of the natural compound Nobiletin (NOB), which directly activates circadian cellular oscillators, showed a reduction in sleep disturbances and an increase in oxygen consumption and CO_2_ production in a female AD mouse model [110]. Additionally, this study indicated that the expression of multiple core clock genes in the cerebral cortex, such as *Bmal1*, Neuronal PAS Domain Protein 2 (*Npas2*), and RAR-related orphan receptor alpha (*Rora*) was altered following the administration of NOB.

Furthermore, in a randomized controlled trial, the effect of donepezil and galantamine at different time points during the day on sleep quality of AD patients was evaluated [111]. This study indicated that patients who took donepezil in the morning, compared to at night, experienced an improvement in sleep quality and decreased daytime drowsiness. These findings indicate that the administration of acetylcholinesterase inhibitors in the morning may enhance the sleep quality of patients with AD.

## 9. Limitations

Narrative reviews have inherent shortcomings of the non-standardized literature search, potential bias in the appraisal of extracted papers, and finding interpretations; nevertheless, they generally provide a smooth and up-to-date reference in specific areas for readers of interest [112]. The present narrative review also carries the aforementioned inherent limitations.

## 10. Conclusions

Circadian rhythms play a crucial role in regulating various biological processes. There is a link between CRDs and AD. These disruptions can also be reflected by structural and functional changes in the SCN of the brain. Sleep problems impair Aβ clearance in the brain and worsen the disease progression. In AD patients, the rhythmic expression of clock genes is hindered, which exacerbates the CRDs. CRDs, in turn, lead to synaptic dysfunction, neuroinflammation, and neurodegeneration, further accelerating the AD progression. Similarly, inflammatory processes in AD appear to follow a circadian pattern, and in turn, a disturbance in core clock genes can lead to neuroinflammation. Although some studies have already addressed the potential of managing CRDs as a supplementary therapeutic approach for AD, more research in this field is needed. Additionally, some of the cited references in our review paper used animal models. Animal models are helpful for the study of AD, but translating findings from animal studies to human pathology is complicated. Rodent models for AD can effectively reflect aspects of Aβ accumulation and tau pathology. Animal models may not fully simulate human neurodegeneration and cognitive impairment. Yet, their findings give an understanding of AD molecular and cellular mechanisms, such as amyloid processing, tau hyperphosphorylation, and neuroinflammation. Future studies to investigate biomarkers of circadian dysfunction that could contribute to early AD diagnosis, such as changes in peripheral expression of clock gene and patterns of melatonin secretion are suggested. A more detailed study on sex differences in CRDs and their impact on AD would contribute to personalized therapeutic strategies.

## Figures and Tables

**Table 1:** Circadian clock gene polymorphisms associated with AD.

Gene	Polymorphism (SNP)	Findings in AD Patients	Reference
*CLOCK*	rs4580704	Higher prevalence of C allele carriers	Chen et al. [44]
*CLOCK*	rs1554483	Higher prevalence of G allele carriers	Chen et al. [45]
*PER1*	rs3027178	G allele is protective against AD	Bacalini et al. [46]
*PER3*	Five repeat homozygotes of *Per3* length	Increased prevalence of five-repeat homozygotes	Xiang et al. [47]
*BMAL1*	rs2278749	Higher prevalence of TT genotypes	Chen et al. [48]
*BMAL2*	rs2306074	Higher prevalence of C allele carriers and CC genotypes	Qing-Xiu et al. [49]
*CLOCK*	3111T/C	Higher prevalence of C allele carriers	Yang et al. [22]
*CLOCK*	rs4864548	Higher prevalence of A allele carriers	Peng et al. [50]
*BMAL1*	rs900147	Higher risk of developing AD in G allele carriers	Li et al. [51]
*PER3*	rs228697	There may be a higher risk of CRDs in APOE ε4 carriers	Lozano-Tovar et al. [52]

Abbreviations: AD, Alzheimer’s Disease; SNP, Single Nucleotide Polymorphisms; CLOCK, Circadian Locomotor Output Cycles Kaput; *PER*, Period; *BMAL*, basic helix-loop-helix ARNT like; CRDs, Circadian Rhythm Disturbances; APOE ε4, Apolipoprotein E4.
